# Proteomic identification of *Drosophila melanogaster *male accessory gland proteins, including a pro-cathepsin and a soluble γ-glutamyl transpeptidase

**DOI:** 10.1186/1477-5956-4-9

**Published:** 2006-05-02

**Authors:** Michael J Walker, Caroline M Rylett, Jeff N Keen, Neil Audsley, Mohammed Sajid, Alan D Shirras, R Elwyn Isaac

**Affiliations:** 1 Faculty of Biological Sciences, University of Leeds, Leeds, LS2 9JT, UK; 2 Environmental Biology Group, Central Science Laboratory, Sand Hutton, York YO41 1LZ, UK; 3 Sandler Center for Basic Research in Parasitic Diseases, Department of Pathology, University of California San Francisco, HSW501 San Francisco, CA 94143, USA; 4 Department of Biological Sciences, University of Lancaster, LA1 4YQ, UK

## Abstract

**Background:**

In *Drosophila melanogaster*, the male seminal fluid contains proteins that are important for reproductive success. Many of these proteins are synthesised by the male accessory glands and are secreted into the accessory gland lumen, where they are stored until required. Previous studies on the identification of *Drosophila *accessory gland products have largely focused on characterisation of male-specific accessory gland cDNAs from *D. melanogaster *and, more recently, *Drosophila simulans*. In the present study, we have used a proteomics approach without any sex bias to identify proteins in *D. melanogaster *accessory gland secretions.

**Results:**

Thirteen secreted accessory gland proteins, including seven new accessory gland proteins, were identified by 2D-gel electrophoresis combined with mass spectrometry of tryptic fragments. They included protein-folding and stress-response proteins, a hormone, a lipase, a serpin, a cysteine-rich protein and two peptidases, a pro-enzyme form of a cathepsin K-like cysteine peptidase and a γ-glutamyl transpeptidase. Enzymatic studies established that accessory gland secretions contain a cysteine peptidase zymogen that can be activated at low pH. This peptidase may have a role in the processing of female and other male-derived proteins, but is unlikely to be involved in the processing of the sex peptide. γ-Glutamyl transpeptidases are type II integral membrane proteins; however, the identified AG γ-glutamyl transpeptidase (GGT-1) is unusual in that it is predicted to be a soluble secreted protein, a prediction that is supported by biochemical evidence. GGT-1 is possibly involved in maintaining a protective redox environment for sperm. The strong γ-glutamyl transpeptidase activity found in the secretions provides an explanation for the observation that glutamic acid is the most abundant free amino acid in accessory gland secretions of *D. melanogaster*.

**Conclusion:**

We have applied biochemical approaches, not used previously, to characterise prominent *D. melanogaster *accessory gland products. Of the thirteen accessory gland secreted proteins reported in this study, six were represented in a *D. simulans *male accessory gland EST library that was biased for male-specific genes. Therefore, the present study has identified seven new secreted accessory gland proteins, including GGT-1, which was not recognised previously as a secreted accessory gland product.

## Background

The male accessory glands (AGs) of *Drosophila melanogaster *are necessary for male fertility [1,2]. They are responsible for the synthesis and secretion of a large number of seminal fluid proteins, many of which have not been structurally or functionally characterised. The most studied AG products are male-specific proteins (Acps) that elicit multiple physiological and behavioural effects in post-mated females [3]. These male influences include increased rate of egg-laying, reduced attractiveness to males, rejection of courting males, better sperm storage and a reduction in female life-span [3]. The altered behaviour of mated females can last up to one week and this long-term effect is dependent upon transfer of both sperm as well as seminal fluid. The sex peptide (Acp70A) is partly responsible for eliciting the short-term female responses and solely responsible for the longer lasting changes in post-mated female behaviour [4,5]. The latter effect is dependent upon the presence of sperm that carry sex peptide bound to the surface of sperm tails [6]. The tethered sex peptide is slowly-released from the sperm by proteolytic cleavage of the N-terminal binding domain from the C-terminal sequence, which contains the signal for the post-mating response [7]. A second AG product (ovulin, ACP26Aa) is also involved in stimulating the early responses in mated females and appears to have a direct effect on ovulation [8-10]. Ovulin also undergoes proteolysis in the female reproductive tract to release biologically active peptides, a process that is dependent upon another, as yet unidentified, male AG product [11]. Sperm storage in the post-mated female requires Acp36DE, a 122 kDa glycoprotein that is processed to a 68 kDa protein in the female within ten minutes of transfer from the male [12-14].

Other seminal fluid proteins that have been studied include peptidase inhibitors, components of the mating plug, lipases and anti-bacterial peptides (reviewed in [3]). An expressed sequence tag screen of a cDNA library prepared from the AGs of *D. simulans *and purposely enriched for male-specific cDNAs, indicated that there might be in total over 70 Acps in this species [15]. Several classes of enzymes, including lipases and peptidases, were well represented in the *D. simulans *AG ESTs, as were peptidase inhibitors and immune defence proteins [16]. This information has led to a re-assessment of the total number of secreted *D. melanogaster *Acps, which is now thought to be 52 [17]. However, this number will exclude AG products, which are highly expressed in female tissues, but are vital for male reproductive success. Specific antibodies raised to thirteen *D. melanogaster *Acps showed that these male AG proteins have multiple fates once transferred to the female reproductive tract, with some proteins entering the female haemolymph [18].

We now report the direct characterisation of protein products of *D. melanogaster *AGs, which were resolved by 2D-gel electrophoresis and identified by mass spectrometry of tryptic peptides. This study identifies thirteen AG proteins, including protein-folding and stress-response proteins, a lipase, a cysteine-rich protein and two peptidases, a pro-form of a cathepsin-like cysteine peptidase and a soluble γ-glutamyl transpeptidase. Seven of these proteins have not previously been identified as secreted AG products. We also show that the AG secretions possess cysteine peptidase and γ-glutamyl transpeptidase activities and that the cysteine peptidase is synthesised primarily as an inactive zymogen. The cathepsin K-like cysteine peptidase is a candidate for the processing enzyme responsible for the delayed cleavage of male proteins and peptides in the uterus of the post-mated female.

## Results and discussion

### Identification of secreted AG proteins

**Figure 1:**
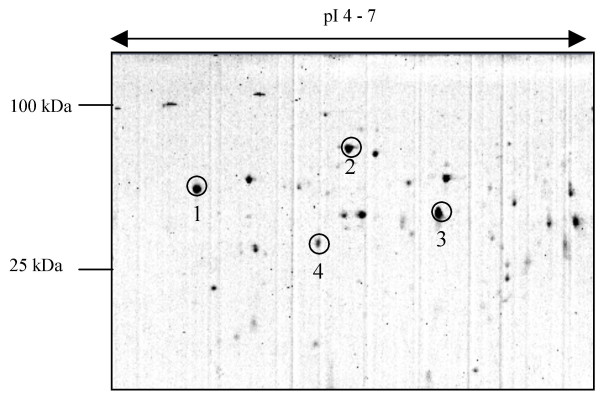
A representative 2-D gel (pH 4–7) stained with Coomassie blue, showing the separation of proteins from 80 male AGs. Protein spots that were unambiguously identified as secreted proteins in this experiment are labelled. 1, CG9429; 2, CG4147; 3, CG4847; 4, CG17575.

AG contents were analysed by 2D-gel electrophoresis; the first dimension used either pH 3–10 or pH 4–7 IPG strips (Fig. 1). The gels were Coomassie stained and the most intense resolved protein spots subjected to trypsin digestion and peptide fragment analysis by either MALDI-MS or LC-MS/MS. Thirteen AG proteins with predicted signal peptides for secretion were identified using this method and their functions were inferred from comparison of the primary structure to members of protein families with known biological activity (Table 1). Almost half of these proteins could be classed as stress response proteins or as proteins required for the efficient and correct folding of newly synthesised proteins in the ER lumen. The other group of proteins contains two peptidases, a lipase, a protein hormone (Acp36DE), a cysteine-rich protein and a serpin.

**Table 1:** *D. melanogaster *accessory gland proteins identified by proteomics. AG proteins with a secretory signal peptide identified by either MALDI-MS or LC-MS/MS of tryptic fragments of AG protein spots excised from 2D gels. Data presented in parenthesis is derived from MALDI-MS analysis

CG number	Protein name	Homology	Predicted function	Number of peptides matched	% coverage	Estimated protein Mr (kDa)	Theoretical Mr (kDa)
CG2852		peptidyl-prolyl cis-trans isomerase	protein folding	11(8)	57 (46)	20	22
CG2918		heat shock protein 70 kDA	chaperone	14	17	106	103
CG4147	Hsc-70-3	heat shock protein	chaperone	(15)	(26)	71	72
CG4847		cathepsin K	peptidase	3 (9)	8 (39)	42	44
CG5520	Gp93	histidine kinase-like ATPase	chaperone	2	3	92	90
CG6461	Ggt-1	γ-glutamyl transpeptidase	peptidase	2	5	24	63
CG6988	Pdi	protein disulfide isomerase	protein folding	8	23	62	56
CG7157	Acp36DE		hormone	(3)	(3)	102	102
CG9334	Spn3	serpin	peptidase inhibitor	(14)	(45)	45	42
CG9429	calreticulin	chaperone	chaperone	(8)	(21)	53	47
CG9847	Fkbp13	peptidyl-prolyl cis-trans isomerase	protein folding	2	12	26	24
CG17575		CRISP		(6)	(25)	33	32
CG31872		triacylglycerol lipase	lipid metabolism	6 (4)	7 (4)	118	119

### Protein folding and stress response proteins

CG2852 (cyclophilin-like) and CG9847 (Fkbp-13) are peptidyl-prolyl *cis-trans *isomerases that catalyse the *cis-trans *isomerisation of peptidyl-prolyl bonds in polypeptide chains. They belong to two different structural classes that are phylogenetically conserved and are known to have multiple roles in the folding, assembly and trafficking of cellular proteins [19]. Although considered to be primarily intracellular proteins, some mammalian cells secrete cyclophilins into the extracellular environment, where they have an important role in mediating cellular responses to oxidative stress and in modulating immunity. CG6988 is a protein disulfide isomerase (PDI) that contains two thioredoxin-like folds and is highly expressed at all stages of development [20]. The PDI family of proteins catalyse the formation, reduction and isomerisation of disulphide bonds and can protect cells from hypoxic cell death [21]. Their ability to bind polypeptide chains allows PDI to act as a molecular chaperone assisting with the folding of polypeptides and increasing the yield of correctly folded proteins.

Four other molecular chaperones (CG9429/calreticulin; CG4147/HSP70-3; CG2918; CG5520/Gp93) were identified in this proteomic study of male AGs. *D. melanogaster *calreticulin belongs to the highly conserved family of lectin-like Ca^2+^-binding ER proteins that have diverse cellular roles in mammals, including regulation of intracellular Ca^2+^, the correct folding of glycoproteins, integrin-mediated Ca^2+ ^signalling, phagocytosis and cell adhesion [22,23]. HSP70-3 is a BiP homologue involved in assembling protein complexes inside the endoplasmic reticulum [24]. Gp93 is a highly conserved heat shock protein (HSP) of the vertebrate gp96 family of endoplasmic reticulum proteins. Galewsky *et al*. have reported that Gp93 accumulates at cell borders suggesting that the *D. melanogaster *protein can be exported from the cell to the extracellular surface [25]. CG2918 is structurally also a member of the HSP family of proteins with strong homology to mammalian hypoxia up-regulated protein ROP150 [26].

Five of the aforementioned AG protein-folding and stress-response proteins possess a K/HDEL C-terminal sequence (the C-terminus of CG2918 is HSEL) suggesting that these proteins are resident in the lumen of the endoplasmic reticulum. However, it is well documented that proteins entering the secretory pathway can escape KDEL-mediated endoplasmic reticulum-retention to have a functional role at the cell surface or in the extracellular milieu [23,27]. Indeed, recent studies have shown that cell-surface chaperone proteins can have important roles in reproduction by functioning as intercellular signalling molecules that facilitate sperm-oocyte interaction [28-30].

### Acp36DE (CG7157), cysteine-rich protein (CG17575) and serpin-3 (spn-3, CG9334)

Acp36DE (CG7157) has been extensively studied and shown to be required for sperm storage [12,14,31], although the mechanism by which this is achieved is not known. CG17575 has been shown by comparative structural modelling to be a member of the CRISP family (cysteine-rich secretory protein) [16]. Interestingly, mammalian CRISP-1 is secreted by the proximal epididymis, binds to the sperm surface, and appears to be involved in gamete fusion [32]. CRISP-2 is secreted from spermatocytes and facilitates enhanced binding of spermatocytes to Sertoli cells. Allurin, another CRISP, acts as a sperm chemoattractant produced by female *Xenopus *as part of the egg jelly [33]. In addition to these suggested roles in reproduction, CRISP proteins of the CRISP-3 class might also form part of the innate immune defence system [34]. Antibodies raised to recombinant CG17575 were used in a recent study to determine the fate of the protein in the mated female. Western blot analyses suggested a broad distribution for CG17575 in female reproductive tissues, including the uterus, oviduct and egg surface [18], consistent with a possible role in gamete fusion.

Two members (Acp76A and Acp62F) of the serpin family were previously known to be present in *D. melanogaster *AG secretions. The proteomic identification of SPN-3 takes the number of *D. melanogaster *AG serpins to three and this number might be an underestimate, since Swanson and colleagues identified six AG serpin genes in *D. simulans*, including the orthologue of *Spn-3 *[15]. The expression of a number of serpin family genes in the AG probably reflects the importance to the male of controlling proteolysis by serine peptidases in the seminal fluid [16]. Evidence for such a role for serpins in animal reproduction comes from studies on male mice lacking the serpins, PCI and nexin-1 [35,36]. These animals display abnormal spermatogenesis and malformation of the copulatory plug presumably as a result of a failure to control peptidase activity. Immunological studies on the fates of *D. melanogaster *Acps in the female, post-copulation, identified CG9334 in both the mating plug and the sperm storage organs [18].

### Acid lipase (CG31872)

The AGs of *D. melanogaster *are known to be a rich source of an acid lipase capable of cleaving triacylglycerol and to some extent cholesterol esters. Smith *et al*. showed that the lipase activity of males was halved after mating, whereas the activity in mated females was three-times that of virgin female flies, indicating transfer in the seminal fluid to the female during copulation [37]. Our proteomics analysis has identified CG31872 as a candidate for this activity. These enzymes might have an important role in providing energy for motile sperm by hydrolysing triglycerides to release free fatty acids for β-oxidation. The work of Mueller *et al*. [17]identified five acid lipase related AG ESTs of *D. simulans *and therefore we might expect additional lipases to be present in the AGs of *D. melanogaster*.

### Procathepsin protein (CG4847) and the demonstration of pro-form of a cysteine peptidase in AG secretions

**Figure 2:**
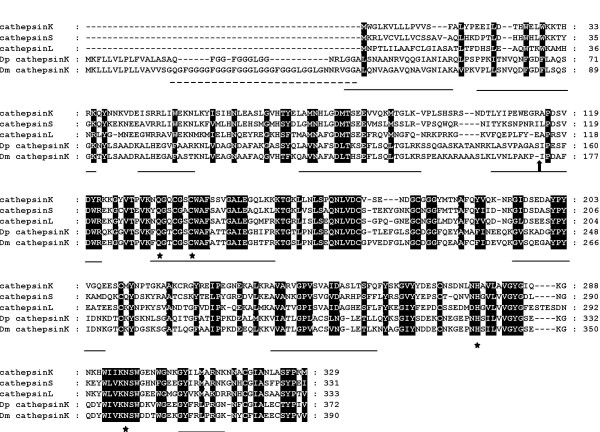
ClustalX amino acid sequence alignment of *D. melanogaster *cathepsin K (Dm cathepsinK, CG4847), *D. pseudoobscura *cathepsin K (Dp cathepsinK, GA18475) and human cathepsin K, S, and L. Active site residues, stars; Gly-rich region, dashed line; predicted cleavage to generate mature cathepsin, filled block arrow; tryptic peptides identified by mass spectrometry, underline. Conserved amino acids are in black.

CG4847 encodes a protein that structurally belongs to the papain (C1A) family of cysteine peptidases and is related to mammalian cathepsin K, L and S (Fig. 2) [38]. CG4847 has a predicted molecular weight of 44 kDa in its unprocessed form and, uniquely for this peptidase family, posesses a Gly-Gly-Gly-Phe/Leu repeat sequence close to the N-terminus of the protein. BLAST search of the predicted proteins of the *Drosophila pseudoobscura *genome identified GA18475 as a close homologue of CG4847 that shares 68% identity at the amino acid level. The predicted *D. pseudoobscura *also has a Gly/Leu-rich region following a signal peptide; however this region is shorter than that in CG4847 (Fig. 2).

Cathepsins are typically synthesised as inactive pro-enzymes that, under favourable conditions, undergo intracellular auto-cleavage that removes an N-terminal pro-domain to generate an active peptidase of around 24 kDa in size [39]. The observation that CG4847 migrates in SDS-polyacrylamide gel electrophoresis with a mass of around 50 kDa and the fact that the peptide mass fingerprint contained five peptides from the predicted pro-domain, indicates that the enzyme is secreted with a typical zymogen structure (Fig. 2). We sought enzymatic evidence for the synthesis and secretion of a pro-cathepsin cysteine peptidase by male AGs. AG contents were incubated in activation buffer, prior to performing a standard assay for C1A cathepsins using the substrate Cbz-Leu-Arg-MCA in a continuous assay for peptidase activity. The hydrolysis of the substrate was linear with time and was strongly inhibited by two irreversible inhibitors of cysteine peptidases (E64 and K11777), all at a concentration of 10 μM (Table 2). Mammalian pro-cathepsin K, like many other cysteine peptidases, undergoes autoactivation as a result of a conformational change on lowering the pH [40]. The induced structural change exposes the pro-cathepsin K active site to increase the susceptibility of the pro-peptide to cleavage. When the activation procedure for the AG cysteine peptidase was performed at 4°C rather than at 35°C, the peptidase activity was greatly reduced (Table 2). This result is consistent with the peptidase being secreted as a zymogen that can be activated in a temperature-dependent manner at low pH. It is possible that the activation of the AG cysteine peptidase occurs after insemination by autocatalysis after experiencing a change in pH in the female reproductive tract or by exposure to another peptidase either present in the seminal fluid or secreted from female reproductive tissues.

**Table 2:** Peptidase activity of *D. melanogaster *accessory glands. Results are expressed as the mean of three experiments ± s.e.m

Enzyme activity	Rate of hydrolysis (pmol of 7-AMC released/h/AG)
Cysteine peptidase	
pre-activation	0.7 ± 0.1
post-activation	4.5 ± 1.3
post-activation + 0.1 μM E64	0.18 ± 0.01
post-activation + 0.1 μM K11777	0.25 ± 0.03

A potential role for the CG4847 is in the proteolytic processing of hormone precursors. The predicted protein is structurally related to mammalian cathepsin L, which in secretory vesicles of chromaffin cells functions as a prohormone processing peptidase responsible for the production of enkephalin peptides [41]. The cathepsin L recognises monobasic and dibasic processing sites with the positively charged side-chains occupying either the S1 or S1' sub-sites. Two *D. melanogaster *AG prohormones, ovulin and sex peptide, undergo proteolytic processing in the post-mated female and in both cases, basic residues are the cleavage recognition sites [7,11]. CG4847 might perform a similar prohormone processing role to cathepsin L and be involved in the cleavage of both ovulin and sex peptide after transfer to the mated female. We have investigated the possibility that the AG cysteine peptidase is responsible for the cleavage of the sex peptide by testing whether the activated enzyme can cleave sex peptide 1–13 (WEWPWNRKPTKFP), which contains two potential prohormone cleavage sites, at the Arg^7^-Lys^8 ^and Lys^11^-Phe^12 ^peptide bonds. HPLC analysis with UV detection (280 nm) identified a single major cleavage product that was identified as WEWPWNRKPTK by MALDI-MS ([M+H]^+ ^*m/z *1528). This hydrolysis was completely blocked in the presence of 10 μM E64, confirming that the AG cysteine peptidase activity was responsible. Peng *et al*. have shown that the N-terminal region of sex peptide is required for sperm attachment and for the long-term post-mating response of females [7]. They have also shown that mutating the Arg^7 ^and Lys^8 ^to Gln^7 ^and Gln^8 ^prevents the release of sex peptide from the surface of the sperm tail over a 5 day period, which suggests that proteolysis occurs at, or close to, the dibasic motif. Since the AG cysteine peptidase activity cleaves Lys^11^-Phe^12^, which is four peptide bonds away from Arg^7^-Lys^8^, it is unlikely that the enzyme has a major role in the processing of the sex peptide in the post-mated female.

### γ-Glutamyl transpeptidase (CG6461, GGT-1) and detection of γ-glutamyl transpeptidase activity in AG secretions

**Figure 3:**
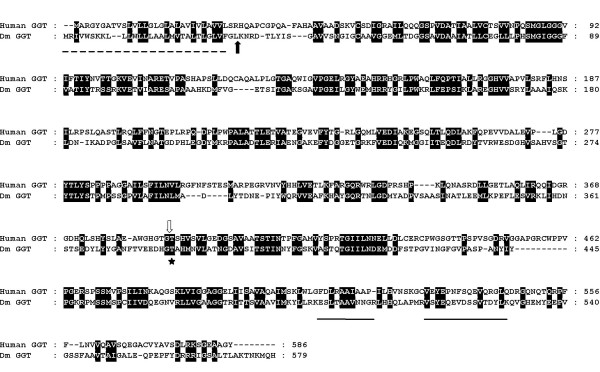
ClustalX alignment of *D. melanogaster *GGT-1 (CG6461) and human GGT-1. Hydrophobic signal sequence/transmembrane anchor, dashed line; predicted cleavage of the signal peptide for GGT-1, filled block arrow; predicted cleavage site necessary for activation, open block arrow ; active site Thr, *; tryptic peptides identified by mass spectrometry, underline. Conserved amino acids are in black.

CG6461(GGT-1) is predicted to be a 62 kDa γ-glutamyl transpeptidase (also known as γ-glutamyl transferase) and a member of the T3 family of threonine peptidases [42]. These enzymes are abundant on the luminal surface of secretory and absorptive epithelial cells and are responsible for the transfer of γ-glutamate from glutathione (GSH), glutathione conjugates and glutathione disulphide (GSSG) to an acceptor molecule, which may be another peptide, an amino acid, or water. The proenzyme undergoes autocatalytic cleavage to generate the active γ-glutamyl transpeptidase, comprising two subunits of around 40 kDa and 20 kDa [43]. The catalytic nucleophile is the hydroxyl group of the threonine found at the N-terminus of the smaller processed subunit [44]. The AG peptide fragments identified in the present study came from a protein spot with an estimated size of around 20 kDa and corresponded to sequences within the predicted smaller subunit of GGT-1 (Fig. 3). The SignalP 3.0 server predicts with high probability that GGT-1 is a preproprotein comprising an N-terminal signal peptide for secretion and that this is cleaved at the Gly^30^-Leu^31 ^bond to generate a soluble, secreted γ-glutamyl transpeptidase [45]. The predicted soluble nature of GGT-1 is unusual for members of this family of enzymes, since eukaryotic γ-glutamyl transpeptidases are typically type II integral membrane proteins found on the external surfaces of cells [42]. There are three other γ-glutamyl transpeptidase genes (CG17636, CG1492 and CG4829) in the *D*. *melanogaster *genome, all of which are predicted by SignalP 3.0 to encode proteins that conform to a membrane-bound structure. In *D. pseudoobscura*, γ-glutamyl transpeptidase genes are fewer in number and the family is represented by one secreted (GA9613) and only one membrane-bound γ-glutamyl transpeptidase (GA13353): these are closely related to *D*. *melanogaster *GGT-1 and CG4829, respectively.

We found strong γ-glutamyl transpeptidase activity (28.1 ± 1.7 μmoles of *p*-nitroanilide released/h/AG; mean ± standard error of the mean, n = 3) in the *Drosophila *AG using γ-glutamyl-*p*-nitroanilide as the substrate donor and the dipeptide, Gly-Gly, as the acceptor. There was a linear increase in enzyme activity, which was absolutely dependent on the presence of Gly-Gly and was inhibited by acivicin (0.4 mM, 98% inhibition), a selective inhibitor of γ-glutamyl transpeptidase. The AG γ-glutamyl transpeptidase activity is not associated with cell membranes since almost all of the activity of the AG homogenate was found in the supernatant after centrifugation at 60,000 ***g ***for 2 h (25.1 ± 2.3 μmoles of *p*-nitroanilide released/h/AG; mean ± standard error of the mean, n = 3).

The identification of a secreted γ-glutamyl transpeptidase in the AG is of particular interest in respect of redox control during reproduction. γ-Glutamyl transpeptidase has a central position in the 'γ-glutamyl cycle', generating glutamate and Cys-Gly by cleavage of GSH. The dipeptide is subsequently cleaved to its constituent amino acids [46], which are taken-up across the plasma membrane and used in the re-synthesis of intracellular GSH. GSH provides reducing equivalents for the reduction of hydrogen peroxide and lipid hydroperoxides by glutathione peroxidases and peroxiredoxins, and is required for detoxification reactions carried out by glutathione S-transferases [47,48]. The maintenance of a high intracellular concentration of GSH is therefore important for preventing injury to the germ cells by reactive oxygen species and xenobiotics, and is highly dependent upon the breakdown and recycling of extracellular GSH compounds [48].

The soluble AG γ-glutamyl transpeptidase activity provides an explanation for the relatively high levels of free glutamic acid known to be present in AG secretions of virgin males of *D. melanogaster *and the rise in uterine glutamate in the post-mated female [49].

## Conclusion

Interest in the male AG products of *D. melanogaster *has mainly focused on the proteins and peptides that are responsible for altering the behaviour and physiology of the post-mated female. There have been few biochemical studies of AG enzymes that are secreted into the seminal fluid for transfer to the female, although their importance for reproduction is recognised. The sequencing of male-specific cDNAs of *D. simulans *AG gave an indication of the variety of new proteins that might be present in the seminal fluid of this species and probably other drosophilids. However, this study was biased against proteins that are expressed in both adult male and female flies, and did not shed light on any post-translational modifications that might be critical for biological activity. We have used the complementary approach of proteomics to expand our knowledge of the secreted AG proteins, mainly enzymes and stress-response proteins, of *D. melanogaster*. We have provided enyzmatic evidence for an inactive cysteine protease and a processed and fully active γ-glutamyl transpeptidase in support of the proteomics data. The cysteine peptidase might undergo activation in the extracellular environment and, if transferred to the female during copulation, might have a role in the processing of male derived proteins (e.g. ovulin) in the mated female. *D. melanogaster *is amenable to reverse genetic approaches, which can now be used to determine the importance and precise role of the identified AG proteins in reproduction.

## Methods

### Materials

Peptidase substrates and inhibitors, unless otherwise stated, were purchased from Sigma-Aldrich Co. Ltd., Poole, U.K. K11777 (*N*-methylpiperazine-urea-phenylalanine-homophenylalanine-vinylsulfone-benzene) was a gift from the Sandler Center for Basic Research in Parasitic Diseases, Department of Pathology, University of California San Francisco.

### Insects

Oregon R *D. melanogaster *were maintained on oatmeal/molasses/agar medium at 25°C with a 12:12 h light/dark regime [50]. For the collection of AG contents for proteomic and enzymatic analysis, males (3–5 days post-eclosion) were separated from females at least 24 h prior to dissection.

### Proteomics

AGs (80 pairs) were excised in ice-cold *Drosophila *Ringer solution [51], or 5 mM Tris-HCl (pH 7.5), containing peptidase inhibitors (complete, Mini, EDTA-free, Roche Diagnostics Ltd, Bell Lane, Lewes, East Sussex BN7 1LG). The AGs were then centrifuged at 10 000 g at 4°C to separate AG contents from the tissue. The supernatant was then removed and stored at -20°C until required. Protein samples were processed using a "2D clean-up kit" (Amersham Biosciences UK Limited, Pollards Wood, Nightingales Lane, Chalfont St Giles, Buckinghamshire). AG contents were solubilised in rehydration buffer (8 M urea, 4% (w/v) CHAPS, 50 mM dithiothreitol, 0.2% (v/v) "Bio-Lyte" Ampholytes, Bio-Rad Laboratories, 2000 Alfred Nobel Drive, Hercules, CA 94547 USA) prior to soaking of either pH 3–10 or pH 4–7 11 cm IPG strip (Bio-Rad). Isoelectric focusing was performed using a PROTEAN IEF cell (Bio-Rad) for 40000 Vh. The strip was then equilibrated in two base buffers (50 mM Tris/Cl, 2% (v/v) SDS, 6 M urea, and 20% glycerol) containing first 1.4 M diothiothreitol and then 1.3 M iodoacetamide. The second dimension used precast 8–16% Tris/HCl gels (Bio-Rad) and was run for 70 min at 200 V. The gel was washed three times for 15 min prior to staining with Coomassie ("biosafe" Bio-Rad) for 3 h before de-staining with water.

Stained spots of protein were cut using a Proteome Works spot-cutter (Bio-Rad) before being placed in 50 mM ammonium bicarbonate and 50 % (v/v) acetonitrile. The gel piece was sonicated for 10 min with the supernatant being removed and discarded. This was repeated before acetonitrile was added and then the gel was dried. The trypsin digest was then carried out by adding 5 μl trypsin solution (2 ng of trypsin in 25 mM ammonium bicarbonate) to the gel piece and allowing it to be absorbed before addition of another 5 μl of 25 mM ammonium bicarbonate 30 min later. Digestion was allowed to proceed for 16 h at 37°C.

MALDI-MS was performed using a matrix of 5 mg of α-cyano-4-hydroxy-cinnamic acid in 1 ml ethanol/acetonitrile (1:1, v/v) with the peptide ACTH used as an internal standard. The matrix was then mixed with the sample in a ratio of 1:1 with 1 μl being spotted onto a MALDI target plate for analysis using MALDI-TOF (Micromass M@LDI L/R system) to generate a peptide mass fingerprint. The NCBI and MSDB peptide databases were searched using Mascot  with, 100 p.p.m. accuracy and carbamidomethyl transformation selected. Some protein spots were analysed by LC-MS/MS and the fragmentation data was used to search the NCBI and MSDB peptide databases using Mascot as described previously [52]

### Enzyme assays

AG contents were separated from the AG tissue by centrifugation in small volumes of Ringer solution, typically 20 pairs of AGs in 40 μl, at 10 000 g at 4°C. For the determination of cysteine peptidase activity, AG samples were incubated in activation buffer (100 mM sodium acetate buffer, pH 5.5; 5 mM EDTA; 20 mM cysteine) at 35°C, either without inhibitor or with one of two cysteine peptidase inhibitors (E64 and K11777). After 1 h, the substrate (Cbz-Leu-Arg-MCA) was added to a final concentration of 10 μM in 1% (v/v) dimethyl sulfoxide and the fluorescence emitted during the reaction was measured every 90 seconds in black 96-well microtitre plates (Corning Life Sciences, High Wycombe, U.K.) at ambient temperature using a Victor^2 ^fluorometer (PerkinElmer™, Finland) and an excitation λ of 380 nm and emission λ of 460 nm. Reaction rates were linear up to 2 h (1 unit of activity is 1 pmol of 7-AMC released/h). Cysteine peptidase activity towards sex peptide1-13 was performed by incubating the contents from 50 AGs in 50 μl of activation buffer for 1 h at 35°C, before adding sex peptide1-13 (50 μM, final concentration). After incubation for 12 h at 35°C, hydrolytic activity was terminated by lowering the pH to 2 with 5 μl of 8% (v/v) TFA. Cleavage products were analysed by reversed phase HPLC using a Phenomenex Jupiter C18 column (5 μm particles, 250 × 4.6 mm; Phenomenex, Macclesfield, U.K.) and an elution gradient of 12% acetonitrile rising to 60% acetonitrile in 0.1% TFA over 25 min, at a flow rate of 1 ml/min. The elution of tryptophan containing peptides was monitored at 280 nm. The major product peak was collected and analysed by MALDI-TOF MS.

*γ-*Glutamyl transpeptidase activity was assayed using γ-glutamyl-*p*-nitroanilide (1 mM) as the γ-glutamyl donor and the dipeptide Gly-Gly (20 mM) as the acceptor in Tris-HCl buffer (0.1 M, pH 8) [42]. The release of *p*-nitroaniline was monitored every 10 sec in 96-well plastic plates (Nunc^TM^, Denmark) using a SPECTRA MAX 340_PC _spectrometer (Molecular Devices, Wokingham, U.K.) set at 410 nm (ε = 8800 M^-1^. cm^-1^) and a temperature of 25°C. The reaction rate was linear up to 1 h (1 unit of activity is 1 nmol of *p*-nitroaniline released/h). The soluble nature of the *γ-*glutamyl transpeptidase was established by homogenising ten AGs in 200 μl of Ringer's solution in a glass homogeniser (GPE Ltd., Leighton Buzzard, UK) and centrifuging the homogenate at 60,000 ***g ***for 2 h in a TLA110 Beckman rotor using an Optima Max Ultracentrifuge (Beckman Coulter UK Ltd, High Wycombe, Buckinghamshire, U.K.). The enzyme activity of resulting soluble fraction was compared to the activity found in a sample of the homogenate taken prior to centrifugation.

## Competing interests

The author(s) declare that they have no competing interests.

## Authors' contributions

MJW, CMR and JNK carried out the proteomic analysis. MJW and MS planned and performed the enzyme activity studies. NA identified the cleavage site for the hydrolysis of sex peptide. REI drafted the manuscript. ADS helped to revise the manuscript and REI and ADS jointly planned the study.
